# Genomic Characterization of Picornaviruses Isolated From Ribbon (*Histriophoca fasciata*) and Harbor (*Phoca vitulina*) Seals

**DOI:** 10.3389/fvets.2020.554716

**Published:** 2020-10-30

**Authors:** Thais C. S. Rodrigues, Ole Nielsen, Kathy A. Burek-Huntington, Vsevolod L. Popov, Stephen Raverty, Dyanna M. Lambourn, Kuttichantran Subramaniam, Thomas B. Waltzek

**Affiliations:** ^1^Department of Infectious Diseases & Immunology, College of Veterinary Medicine, University of Florida, Gainesville, FL, United States; ^2^Department of Fisheries & Oceans Canada, Winnipeg, MB, Canada; ^3^Alaska Veterinary Pathology Services, Eagle River, AK, United States; ^4^Center for Biodefense and Emerging Infectious Diseases, Institute for Human Infections and Immunity, University of Texas Medical Branch, Galveston, TX, United States; ^5^Animal Health Center, Abbotsford Agriculture Centre, Provincial Government of British Columbia, Abbotsford, BC, Canada; ^6^Marine Mammal Investigations, Washington Department of Fish and Wildlife, Lakewood, WA, United States

**Keywords:** aquamavirus, harbor seal, marine mammal, pathogen discovery, picornaviridae, pinniped, ribbon seal, virus

## Abstract

The seal picornavirus 1, species *Aquamavirus A*, is currently the only recognized member of the genus *Aquamavirus* within the family *Picornaviridae*. The bear picornavirus 1 was recently proposed as the second species in the genus under the name aquamavirus B. Herein, we determined the complete genomes of two novel pinniped picornaviruses, the harbor seal picornavirus (HsPV) and the ribbon seal picornavirus (RsPV). The HsPV and the RsPV were isolated in Vero.DogSLAMtag cells from samples collected from stranded harbor (*Phoca vitulina*) and ribbon (*Histriophoca fasciata*) seals. RsPV-infected Vero.DogSLAMtag cells displaying extensive cytopathic effects were processed for transmission electron microscopy and revealed non-enveloped viral particles aggregated into paracrystalline arrays in the cytoplasm. A next-generation sequencing approach was used to recover the complete genomes of the HsPV and the RsPV (6,709 and 6,683 bp, respectively). Phylogenetic and genetic analyses supported the HsPV and the RsPV as members of the *Aquamavirus* genus. Based on these results, RsPV represents a novel strain of *Aquamavirus A*, while the HsPV is a novel strain of the proposed species aquamavirus B. These discoveries provide information on the evolutionary relationships and ultrastructure of aquamaviruses and expands the known host range of those viruses. Our results underscore the importance of the application of classical virology and pathology techniques coupled with high-throughput sequencing technologies for the discovery and characterization of pathogens in wild marine mammals.

## Introduction

The family *Picornaviridae* (order *Picornavirales*) is a diverse assemblage of viruses that possess small spherical nucleocapsids and positive-sense RNA genomes ranging between 7 and 8.8 kb in size (1, 2). On February 2019, the family included 110 recognized species organized into 47 genera [(3); http://picornaviridae.com/]. Picornaviruses have been described from a wide range of hosts, including fish, amphibians, reptiles, birds, humans, and a variety of other mammals including marine mammals. Picornaviruses may cause subclinical to severe infections in humans and animals, such as febrile illness and diseases of the heart, liver, respiratory tract, gastrointestinal tract, and central nervous system (3, 4). They cause diseases of significance to public health including the common cold [most commonly caused by rhinovirus A, B, and C; (5)], poliomyelitis [caused by poliovirus; (6)], and hepatitis A [caused by hepatovirus A; (7)]. Coxsackie A16 and enterovirus 71 are commonly associated with hand, foot, and mouth disease but may also result in aseptic meningitis, encephalitis, myocarditis, or poliomyelitis-like paralysis (8). Enterovirus 76 is associated with diseases including aseptic meningitis, encephalitis, and myocarditis (9).

Picornaviruses are also the cause of notable veterinary diseases including foot-and-mouth disease, the first animal virus ever identified in animals and the cause of a highly contagious disease of livestock (10). In addition, the avian encephalomyelitis virus causes a severe disease in poultry that includes paralysis, ataxia, and muscular dystrophy (11, 12). In marine mammals, nearly all picornaviruses have been reported in pinnipeds including: Sub-Antarctic fur seals *Arctocephalus tropicalis* [fur seal sakobuvirus and fur seal picorna-like virus; (13)], South American fur seals *Arctocephalus australis* [fur seal picornavirus; (13)], harbor seals *Phoca vitulina* [phopivirus, species *Hepatovirus B;* (14)], California sea lions *Zalophus californianus* [*California* sea lion sapelovirus 1 and 2; (15)], and ringed seals *Phoca hispida* (seal picornavirus 1 (SePV-1), *Aquamavirus A*) (16). A single report of a picornavirus has appeared involving a cetacean, the bottlenose dolphin *Tursiops truncatus* [bottlenose dolphin enterovirus; (17)]. The role of picornaviruses in diseases of marine mammals remains to be determined as does their host range, transmission, and prevalence. The complete genome sequence of the SePV-1 was determined from an isolate obtained from the nasal swab of a ringed seal (16). The SePV-1 is the type species (*Aquamavirus A*) and only accepted member of the genus *Aquamavirus* [(3); http://picornaviridae.com/].

Recently, the complete genome sequence of a bear picornavirus 1 (BePV-1) was determined from the visceral organs (lung, spleen, liver, heart and lymph nodes) of an Asiatic black bear *Ursus thibetanus* in northeast China (18). BePV-1 was found to be most closely related to the SePV-1 and was proposed as a second aquamavirus under the proposed name of aquamavirus B. In this study, we isolated picornaviruses from ribbon and harbor seals, sequenced the complete picornavirus genomes, and performed phylogenetic/genetic analyses that demonstrated these pinniped picornaviruses represent novel aquamavirus strains.

## Materials and Methods

### Case Histories and Sample Collection

Three male harbor seals stranded along the shores of Puget Sound, Washington State, USA, from January to February 2008. There were two weaned pups (~6-month-old; animal IDs: 2008-012 and 2008-016) and an adult (16-year-old; animal ID: 2008-010). Additionally, a pregnant adult ribbon seal *Histriophoca fasciata* (~3-year-old; animal ID: 2015-102) was found dead on Adak Island, Alaska, USA, in October 2015. The carcasses were collected by authorized responders of Marine Mammal Stranding networks in Washington and Alaska and submitted for necropsy. Tissue samples were collected using established protocols (19), fixed in 10% buffered formalin, and processed for routine histopathological examination. An additional set of tissues was frozen for virus isolation.

### Virus Isolation and Transmission Electron Microscopy (TEM)

The frozen tissue samples collected were processed for virus isolation using Vero.DogSLAMtag cells as previously described (20). Morbillivirus infection was initially suspected in some of the seals submitted for testing, therefore Vero.DogSLAMtag cell line was used to increase the chances of isolating any morbilliviruses that might be present. This cell line expresses the universal morbillivirus cell receptor signaling lymphocyte activating molecule (SLAM) that allows efficient morbillivirus isolation (21). Flasks were incubated at 37°C and examined daily for cytopathic effects (CPE). Cells were passaged weekly (1:2) for 4 weeks, at which time flasks not showing visible CPE were discarded. Media from flasks showing CPE was passed through a 0.45 μm filter (Millex-HP Syringe Filter Unit, 0.45 μm, Sigma-Aldrich, Oakville, Canada), diluted (1/100) and passaged onto fresh cells. After CPE was observed, the presumed virus-infected Vero.DogSLAMtag culture inoculated with the ribbon seal (2015-102) tissue homogenate was processed for TEM as previously described (22). ImageJ2 software (23) was used to measure the mean diameter and standard deviation of 60 virus particles.

### Genome Sequencing

Vero.DogSLAMtag cell cultures inoculated with tissue homogenates from harbor seals or ribbon seal that exhibited extensive CPE were centrifuged at 5,509 × *g* at 4°C for 20 min using a Beckman JA-14 fixed angle rotor to remove cell debris. Virus particles were then pelleted from the clarified supernatants by ultracentrifugation at 100,000 × *g* at 4°C for 60 min in a Beckman Type 50.2 Ti rotor. Viral RNA was extracted from each of the pellets using a QIAamp Viral RNA Mini Kit (Qiagen, Valencia, USA) according to manufacturer's instructions. Separate cDNA libraries were generated for each of the RNA extracts using a NEBNext Ultra RNA Library Prep Kit (Illumina, San Diego, USA) and sequenced on an Illumina MiSeq sequencer. The resulting paired-end reads were *de novo* assembled using CLC Genomics Workbench (version 9.5.1; Qiagen, Valencia, USA). BLASTX searches of the resulting contigs against a custom virus database, created from virus protein sequences retrieved from the UniProt Knowledgebase (https://www.uniprot.org/uniprot/), were conducted using CLC Genomics Workbench. The integrity of the assembled genome sequences was verified by mapping the reads to the consensus sequences and inspecting the alignments in CLC Genomics Workbench using a window size of 1 bp. The cleavage sites within the polyproteins of the resulting pinniped picornaviruses were predicted by sequence alignment comparisons to the polyproteins of the SePV-1 and BePV-1.

### Phylogenetic and Genetic Analyses

A phylogenetic analysis was conducted by first aligning the amino acid (aa) sequences of the RNA-dependent RNA polymerase proteins (3Dpol) of the pinniped picornaviruses recovered in this study to the orthologous sequences from the type species of each of the 47 recognized genera within the family *Picornaviridae*, all other available pinniped picornaviruses, and the BePV-1. The aa sequence alignment was performed using Geneious Prime 2019.2.1 (https://www.geneious.com) with the multiple alignment using fast Fourier transform (MAFFT) option implemented. A Maximum Likelihood (ML) phylogenetic analysis was performed using IQ-TREE (http://iqtree.cibiv.univie.ac.at/) software with 1,000 non-parametric bootstraps. The aa sequences of the P1, 2C, 3C, and 3D regions of the pinniped picornaviruses recovered in this study were compared to the aquamaviruses (SePV-1 and BePV-1) and two kunsagiviruses (*Kunsagivirus A*; GenBank accession no. NC_038317 and *Kunsagivirus C*; GenBank accession no. NC_034206), the closest relatives to aquamaviruses. These genetic pairwise comparisons were performed using the Sequence Demarcation Tool v1.2 (24) with the MAFFT option implemented.

## Results

### Pathology Findings

Significant lesions observed in the three harbor seals included: verminous pneumonia (*n* = 2), verminous gastroenteritis (*n* = 3), verminous hepatitis (*n* = 1), thrombosis (*n* = 1), bronchointerstial pneumona (*n* = 1), pyelonephritis (*n* = 1), encephalitis (*n* = 3), lymphoid hyperplasia (*n* = 3), and emaciation (*n* = 3). Animal 2008-012 was co-infected with a mammalian orthoreovirus 5 that was isolated from its brain (Nielsen et al., in preparation). Significant pathological findings reported for the ribbon seal included generalized chronic proliferative dermatitis and folliculitis, degenerative myopathy, marked generalized organ congestion, lymphadenopathy, hepatic necrosis, gastrointestinal helminthiasis, verminous pneumonia, and emaciation. The immediate cause of death was attributed to acute severe pigmentary nephrosis, consistent with hemolytic crisis and hemoglobinuria.

### Virus Isolation and TEM

**Figure 1:**
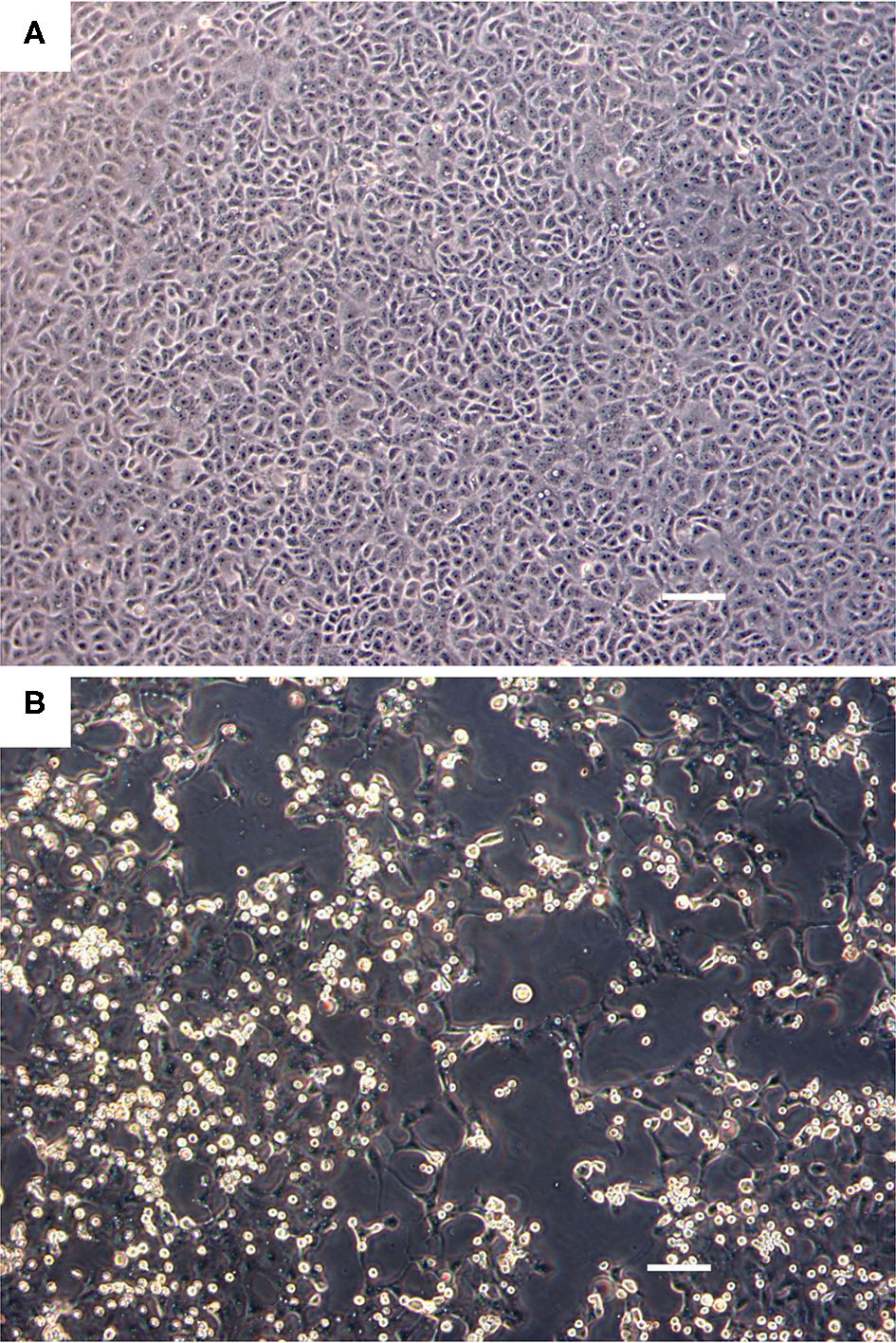
Cell culture images of ribbon seal picornavirus (RsPV) infected Vero.DogSLAMtag cells. **(A)** Uninfected cells. Scale bar = 100 μm. **(B)** Vero.DogSLAMtag cells inoculated with ribbon seal kidney passaged isolate 6 days post-infection showing generalized cytopathology with rounded, enlarged, and refractile cells. Scale bar = 100 μm.

**Figure 2:**
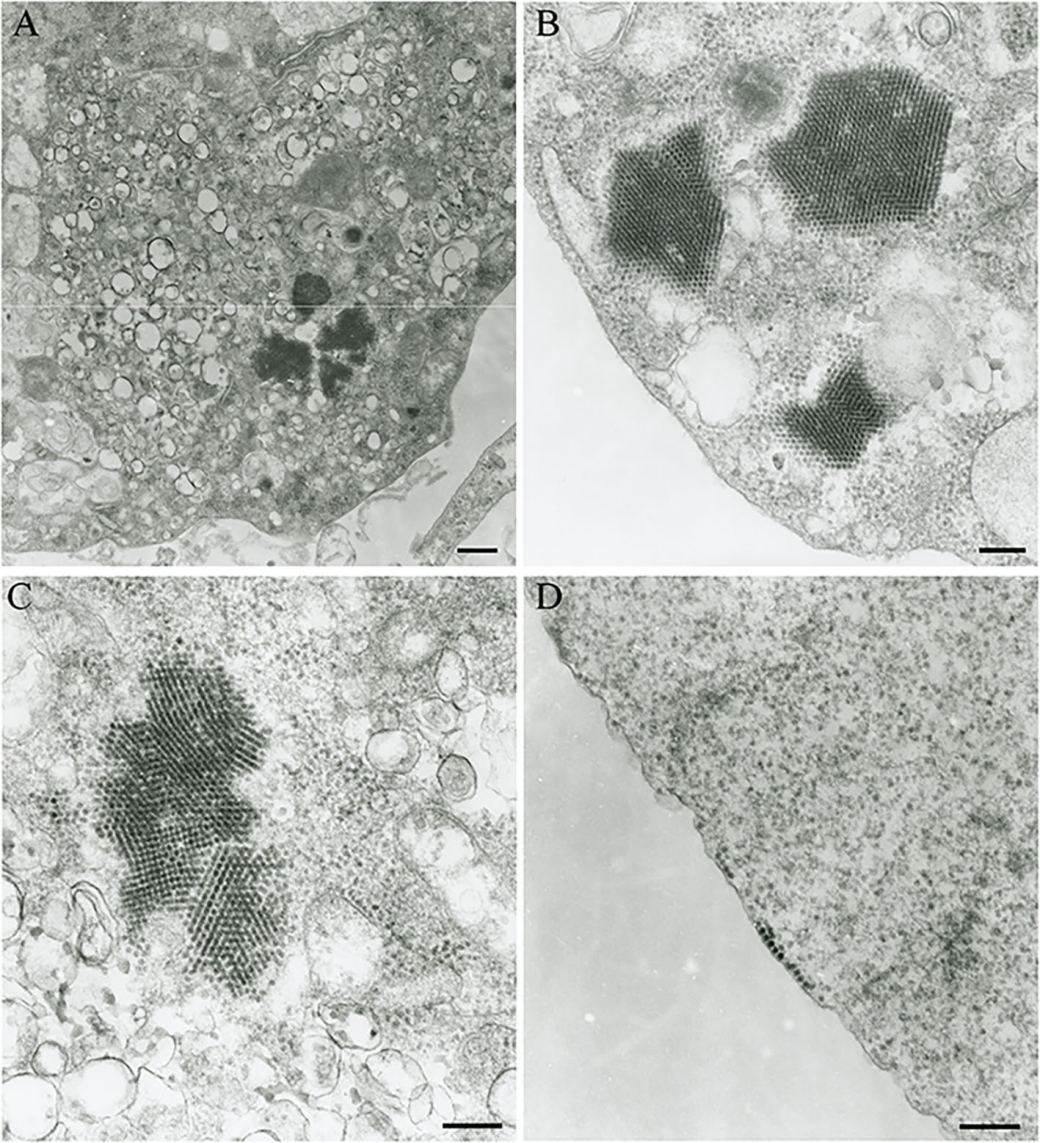
Transmission electron micrograph of Vero.DogSLAMtag cells infected with the ribbon seal picornavirus. **(A)** Portion of the cytosol of an infected cell with multiple vesicles 150–380 nm in diameter and clusters of virus particles packed in paracrystalline arrays. Scale bar = 500 nm. **(B)** Non-enveloped round to icosahedral virions measuring an average of 19.9 nm in diameter within the cells cytoplasm, forming polygonally shaped paracrystalline arrays. Scale bar = 200 nm. **(C)** Higher magnification of the vesicles and paracrystalline arrays within the cytosol. Scale bar = 200 nm. **(D)** Virus particles grouping at the cell membrane prior to budding. Scale bar = 200 nm.

The Vero.DogSLAMtag cultures inoculated with harbor seals spleen (animal 2008-012), mediastinal lymph node (animal 2008-016), and brain (animal 2008-010) tissues developed CPE after three blind passages. Cells became granular and developed a refractile appearance, began to round up, detach from the plastic substrate, and coalesced into clumps, which progressed over a few days until the entire cell sheet was involved. The cells inoculated with the ribbon seal rectal swab and kidney tissue homogenates developed CPE much faster. Within 6 days post-inoculation (dpi), generalized rounding and lifting of infected cells were observed and progressed rapidly with the complete destruction of the cell monolayer by 7 dpi (Figure 1). The flask inoculated with the ribbon seal rectal swab was selected for TEM and showed multiple vesicles within the cytoplasm of Vero.DogSLAMtag cells, along with virus particles aggregated into polygonal paracrystalline arrays (Figure 2). The mean diameter (±SD) of the virus particles was 19.9 **±** 1.8 nm.

### Genome Sequencing

**Figure 3:**
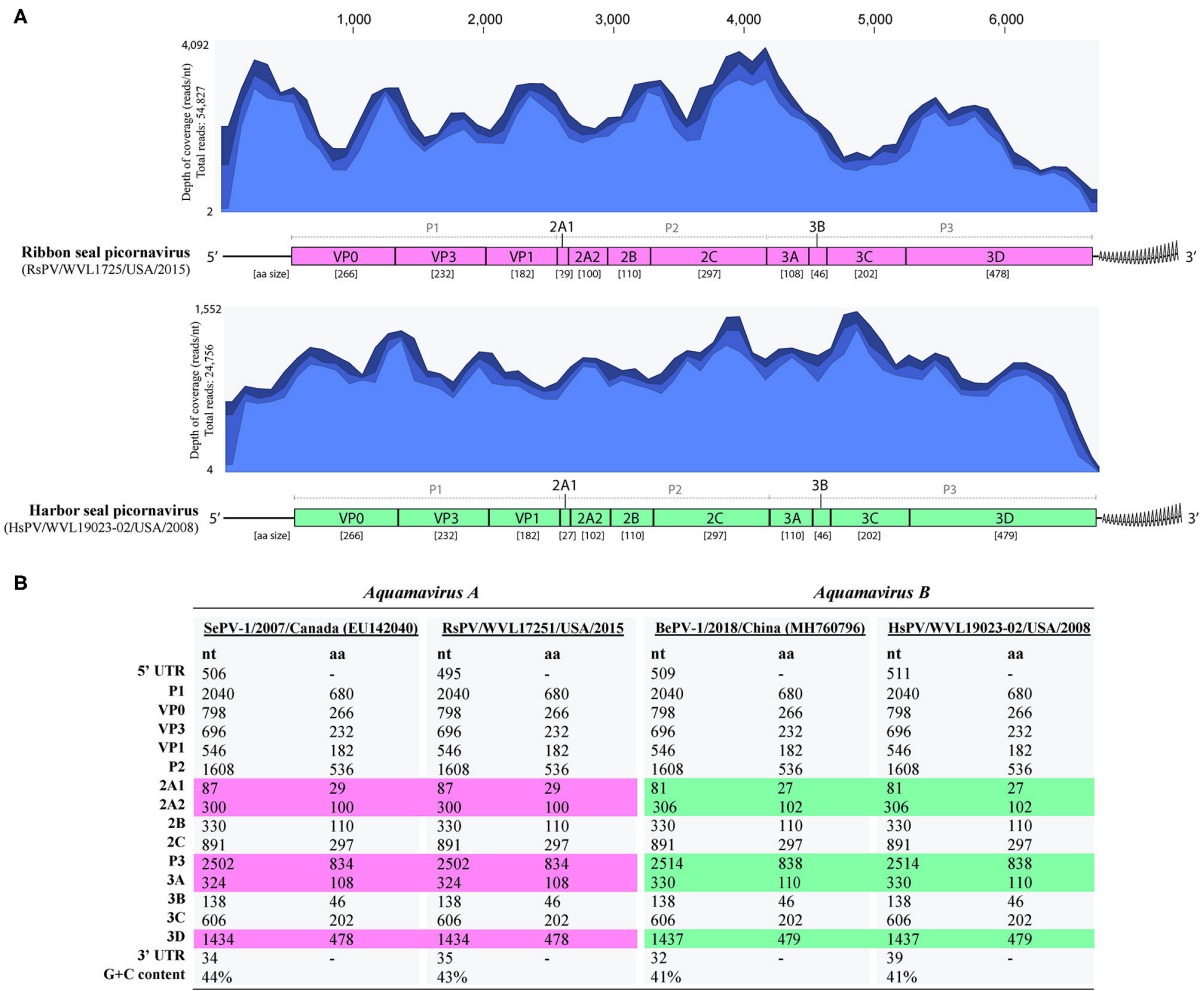
Genomic organization of the harbor seal picornavirus (HsPV) and ribbon seal picornavirus (RsPV). **(A)** Genomic arrangement of HsPV and RsPV depicting the predicted functional domains within the polyproteins and their amino acid (aa) sizes alongside the coverage maps of next-generation sequencing data to the assembled genomes. Different shades of blue from top to bottom on coverage maps show the maximum, average, and minimum coverage values as calculated using a window size of 1 bp. **(B)** Comparison of nucleotide (nt) and aa sizes and G+C content of viruses proposed as species *Aquamavirus A* and aquamavirus B, within genus *Aquamavirus*. Different colors highlight the differences in the size of genome regions between the viruses.

The complete genomes of a picornavirus (hereafter referred to as harbor seal picornavirus; HsPV) were recovered from the cell cultures inoculated with harbor seal spleen (animal 2008-012), mediastinal lymph node (animal 2008-016), and brain (animal 2008-010). The three HsPV isolates were nearly identical and shared >99% nucleotide (nt) identity to each other (data not shown). The genome sequence of the HsPV isolate recovered from the mediastinal lymph node was selected for further genomic analyses. The HsPV genome was determined to be 6,709 bp, with 41% G+C content, excluding the poly(A) tail. Mapping of the assembled HsPV genome resulted in a total read count of 24,756 reads with an average coverage of 1,552 reads/nt (Figure 3A). The HsPV was found to encode a 2,053 aa polyprotein. The genome sequence of HsPV was deposited into the National Center for Biotechnology Information (NCBI) GenBank database under the accession no. MT235242.

The complete genomes of a picornavirus hereafter referred to as ribbon seal picornavirus (RsPV) were recovered from the ribbon seal kidney and rectal swab cell culture suspensions. The RsPV isolates were nearly identical (>99% nt identity) and the RsPV genome sequence recovered from the kidney isolate was chosen for further genomic analyses. The RsPV genome was determined to be 6,683 bp, with 43% G+C content, excluding the poly(A) tail. Mapping of the assembled RsPV genome resulted in a total read count of 54,827 reads with average coverage of 4,092 reads/nt (Figure 3A). The RsPV was found to encode a polyprotein of 2,049 aa, slightly smaller than the polyprotein encoded by HsPV. The genome sequence of RsPV was deposited into the NCBI GenBank database under the accession no. MT235243.

Both HsPV and RsPV were predicted to possess a 3-4-4 picornavirus genome layout: 5′UTR-P1(VP0-VP3-VP1)-P2(2A1-2A2-2B-2C)-P3(3A-3B-3C-3D)-3′UTR. Differences in genomic organization between HsPV and RsPV included the sizes of the predicted proteins 2A1, 2A2, 3A, and 3D (Figure 3B). For both HsPV and RsPV, Walker A GxxGxGKS motifs were identified in the 2C gene region (GAPGSGKS, aa 1045-1052 in both polyproteins) and their putative 3C protease regions included GxCGx_10−15_GxH motifs (GMCGGLLVGKVDGTFKALGFH, aa 1514-1534 in HsPV; GMCGGLLVGKIDGAFKALGFH, 1512-1532 aa in RsPV). The 3D proteins of both viruses include conserved KDE (aa 1730-1732 in HsPV and aa 1728-1730 in RsPV), DxxxxD (DFSAYD; aa 1804-1809 in HsPV and aa 1802-1807 in RsPV), PSG (aa 1860-1862 in HsPV and aa 1858-1860 in RsPV), YGDD (aa 1895-1898 in HsPV and aa 1893-1896 in RsPV), and FLKR (aa 1941-1944 in HsPV and aa 1939-1942 in RsPV) motifs.

### Phylogenetic and Genetic Analyses

**Figure 4:**
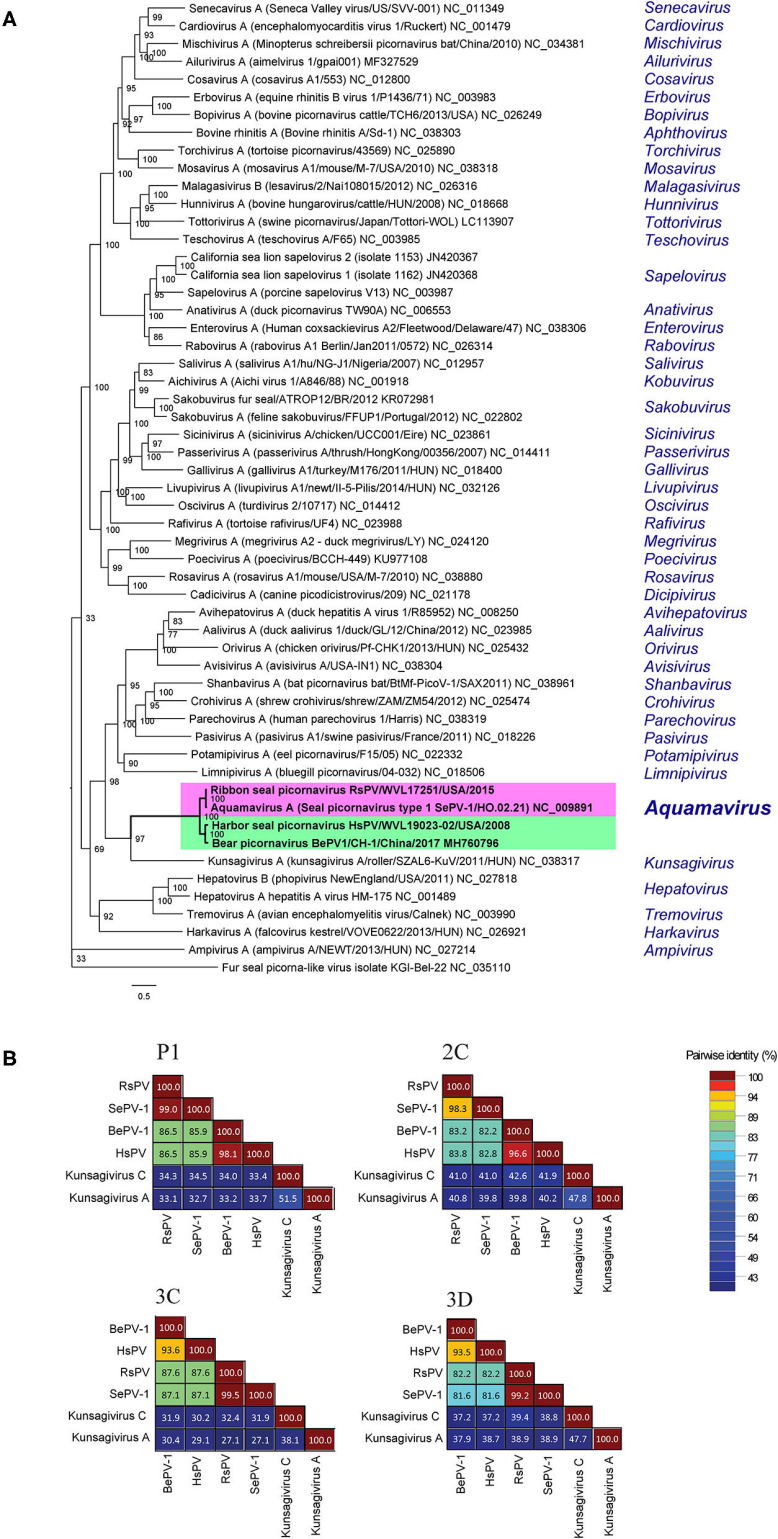
Genetic and phylogenetic analyses of the harbor seal picornavirus (HsPV) and the ribbon seal picornavirus (RsPV). **(A)** Phylogram depicting the relationship of the HsPV and RsPV, within genus *Aquamavirus*, to the type species of all 47 genera within family *Picornaviridae*, including all picornaviruses described from pinniped species. The Maximum Likelihood tree was generated based on the alignment of the amino acid (aa) sequences of the complete 3D gene, using 1000 bootstraps. Branch lengths are based on the number of inferred substitutions, as indicated by the scale. Species name, virus name, and isolate/strain identification are listed of the viruses used are listed (when available), followed by the GenBank accession or RefSeq numbers. The genus within each virus is classified is shown in blue. **(B)** Sequence identity matrices based on the aa alignment of the P1, 2C, 3C, and 3D proteins of the HsPV and the RsPV compared to the most closely related picornaviruses, the seal picornavirus 1 (SePV-1/2007/Canada; EU142040; SePV-1) and the bear picornarvirus (BePV-1/2018/China; GenBank accession no. MH760796; BePV-1), and two related picornavirus used as out-groups: Kunsagivirus A (GenBank accession no. NC_038317) and Kunsagivirus C (GenBank accession no. NC_034206).

The ML phylogenetic analysis supported HsPV and RsPV as members of the genus *Aquamavirus*. HsPV was supported as the sister group to BeHV-1 and RsPV was supported as the closest relative to SePV-1 (Figure 4A). The P1, 2C, 3C, and 3D aa identities between HsPV and RsPV ranged from 82.2 to 87.6%. HsPV showed the highest aa identity to BePV-1 (93.5–98.1%), while RsPV showed the highest aa identity to SePV-1 (98.3–99.5%) (Figure 4B).

## Discussion

The RsPV virion ultrastructure (mean virion diameter = 19.9 nm) and virion morphogenesis were mostly consistent with picornaviruses infecting animals and humans, such as enteroviruses (25) and cardioviruses (26). Picornavirus virions typically possess a diameter of 30–32 nm [*Aphthovirus, Cardiovirus, Hepatovirus, Enterovirus, Parechovirus, Kobuvirus, Senecavirus*; (1, 3)], but may appear smaller in electron micrographs due to drying and flattening during processing (27). Furthermore, a picornavirus measuring 20.0–21.7 nm in diameter has been described from clownfish (*Amphiprion ocellaris*) (28). The cytoplasmic vesicles of infected RsPV Vero.DogSLAMtag cells presumably represent picornavirus replication complexes (29).

HsPV and RsPV possess smaller genomes than most picornaviruses as has been reported for the aquamaviruses SePV-1 (6,693 bp, RefSeq no. NC_009891) and BePV-1 (6,703 bp, GenBank accession no. MH760796). The genomes of HsPV and RsPV exhibit low G+C content when compared to other picornaviruses (3). Similarly, BePV-1 and SePV-1 possess low G+C genomes (41 and 44%, respectively) and present a 3-4-4 picornavirus genome layout. The predicted proteins encoded by HsPV were the same size as those proteins predicted for BePV-1, while RsPV encoded proteins with the same size as the proteins encoded by SePV-1 (Figure 3B).

According to the ICTV *Picornaviridae* Study Group, members within a genus are expected to cluster together as a monophyletic group. In addition, members of different genera usually show significant divergence of orthologous proteins exceeding 66% for P1, 64% for 2C, 3C, and 3Dpol (3). No clear criterion is available for picornavirus species demarcation, but different strains of a species are expected to share a significant identity of P1, 2C, 3C, and 3D proteins and form a monophyletic group in phylogenetic analyses (3).

The presented G+C content, analyses of the polyprotein cleavage pattern and the resulting protein sizes, and phylogenetic/genetic analyses support RsPV as a novel strain of *Aquamavirus A*. The SePV-1 was identified by virus isolation and PCR in samples of lungs, lymph nodes, and nasal swabs of ringed seals from the Beaufort Sea, Canada (16). The geographic range of ribbon seals and ringed seals partially overlap with both inhabiting the Northern Pacific Ocean and Arctic Sea (30, 31). This partial sympatry may have facilitated the spread of similar aquamavirus strains between seal species.

Similarly, our analyses supported HsPV as a novel strain of the proposed species aquamavirus B. Harbor seals are one of the most cosmopolitan pinnipeds and inhabit coastal waters of the northern hemisphere, from temperate to polar regions (32, 33). Another picornavirus was previously described from harbor seals, the phopivirus, and classified as a strain of *Hepatovirus B* within the *Hepatovirus* genus (14). Genetic and phenotypic characterization of the phopivirus revealed high similarity to the hepatitis A virus (HAV; species *Hepatovirus A*), which is the causative agent of an important viral hepatitis in humans. The discovery of phopivirus provided insight into the origin and evolutionary history of HAV-like viruses (14). Likewise, the discovery of HsPV, a closely related strain of the proposed aquamavirus B in an Asiatic black bear [BePV-1; (18)], suggests spillover of aquamaviruses between mammals of the order carnivora may have occurred. Increased surveillance and studies exploring the viromes of wildlife species, including marine mammals, are needed to gain a better understanding of the host range and transmission of aquamaviruses.

Aquamaviruses (SePV-1, BePV-1, RsPV, and HsPV) have been detected in the internal organs (e.g., lungs, lymph nodes, spleen, liver, heart, kidney, and brain) of pinnipeds and the Asiatic black bear. This suggests that aquamaviruses result in systemic infections (16, 18). Although picornaviruses are known to cause disease in a variety of mammalian hosts (34), the contributions of RsPV and HsPV (if any) to the reported pathology of the ribbon and harbor seals was not determined. SePV-1 was highly prevalent in arctic ringed seals hunted from 2000–2002 in Canada, which could represent a recent spread or a more stable endemic relationship of the virus within that seal population (16). The animals from which SePV-1 was isolated appeared healthy (16) and no clinicopathological information was provided on the bear infected with BePV-1 (18). Although many picornaviruses are transmitted horizontally via fecal-oral or airborne routes, transmission of aquamaviruses remains to be determined (3). The isolation of SePV-1 and RsPV from nasal and rectal swabs (16) suggests that both fecal-oral and airborne routes of transmission may be utilized as observed in other picornaviruses.

In this investigation, we report the complete genome sequences of two novel pinniped aquamaviruses. The *in vitro* characteristics, virion ultrastructure and morphogenesis, and genetic/phylogenetic analyses supported RsPV and HsPV as novels strains of *Aquamavirus A* and aquamavirus B, respectively. The results of this study add to a growing body of literature on aquamaviruses and underscores the need for additional research to determine their host range, route(s) of transmission, prevalence, and pathogenicity to terrestrial and aquatic wildlife.

## Data Availability Statement

The datasets presented in this study can be found in online repositories. The names of the repository/repositories and accession number(s) can be found in the article/supplementary material.

## Ethics Statement

The animal study was reviewed and approved by Washington Department Fish Wildlife 109h authority, West Coast Marine Mammal Stranding Network, Alaska Department of Fish and Game (ADF&G) Marine Mammal Permit 15324, NOAA Marine Mammal Stranding Agreement, SR Co-investigator under Permit 932-1905/MA-009526 issued under Section 104 (16 U.S.C. 1375).

## Author Contributions

TR: writing of the original draft manuscript, investigation, formal analysis, and data curation. ON, KB-H, VP, SR, and DL: review and editing of the manuscript, investigation, data curation, and funding acquisition. KS: conceptualization, investigation, formal analysis, software, review, and editing of the manuscript. TW: conceptualization, supervision, funding acquisition, review, and editing of the manuscript. All authors contributed to the article and approved the submitted version.

## Conflict of Interest

KB-H was owner of the company Alaska Veterinary Pathology Services which is a for-profit diagnostic company. Diagnostic analysis was performed under a contract, however there was no monetary compensation for the production of this manuscript. The remaining authors declare that the research was conducted in the absence of any commercial or financial relationships that could be construed as a potential conflict of interest.
